# Changing trends in intestinal parasitic infections among long-term-residents and settled immigrants in Qatar

**DOI:** 10.1186/1756-3305-3-98

**Published:** 2010-10-14

**Authors:** Marawan A Abu-Madi, Jerzy M Behnke, Sanjay H Doiphode

**Affiliations:** 1 Department of Health Sciences, College of Arts & Sciences, Qatar University, P.O. Box 2713, Doha, Qatar; 2 School of Biology, University of Nottingham, University Park, Nottingham, NG7 2RD, U.K; 3 Department of Laboratory Medicine and Pathology, Hamad Medical Corporation, Qatar, P.O. Box 3050, Doha, Qatar

## Abstract

**Background:**

The rapid socio-economic development in Qatar in the last two decades has encouraged a mass influx of immigrant workers, the majority of whom originate from countries with low socio-economic levels, inadequate medical care and many are known to carry patent intestinal helminth and protozoan infections on arrival in Qatar. Some eventually acquire residency status but little is known about whether they continue to harbour infections.

**Methods:**

We examined 9208 hospital records of stool samples that had been analysed for the presence of intestinal helminth and protozoan ova/cysts, over the period 2005-2008, of subjects from 28 nationalities, but resident in Qatar and therefore not recent arrivals in the country.

**Results:**

Overall 10.2% of subjects were infected with at least one species, 2.6% with helminths and 8.0% with protozoan species. Although hookworms, *Ascaris lumbricoides*, *Trichuris trichiura *and *Hymenolepis nana *were observed, the majority of helminth infections (69%) were caused by hookworms, and these were largely aggregated among 20.0-39.9 year-old male subjects from Nepal. The remaining cases of helminth infection were mostly among Asian immigrants. Protozoan infections were more uniformly spread across immigrants from different regions when prevalence was calculated on combined data, but this disguised three quite contrasting underlying patterns for 3 taxa of intestinal protozoa. *Blastocystis hominis*, *Giardia duodenalis *and non-pathogenic amoebae were all acquired in childhood, but whereas prevalence of *B. hominis *rose to a plateau and then even further among the elderly, prevalence of *G. duodenalis *fell markedly in children aged 10 and older, and stayed low (< 2%) gradually falling even further in the elderly. In contrast the prevalence of non-pathogenic amoebae (*Entamoeba coli*, *E. hartmanni*, *Endolimax nana *and *Iodamoeba buetschlii*) peaked in the 30.0-39.9 age group and only then dropped to very low values among the oldest subjects examined. A worrying trend in respect of both helminth and protozoan parasites was the increase in prevalence over the period 2005-2008, in helminth infections prevalence increasing 2-3 fold by 2008, and in protozoan infections by 1.5-2.0 fold.

**Conclusions:**

We suggest that helminth infections are probably acquired abroad when immigrants visit their home villages, whilst protozoan infections are reinforced by transmission in Qatar, possibly in the poorer areas of the state where immigrant workers live. We discuss the significance of these findings and emphasize that they have clear implications for the health authorities.

## Background

In common with other Gulf States, Qatar has experienced rapid socio-economic development in the last two decades, especially in the capital Doha and this has encouraged a mass influx of immigrants to the city, mostly to work in essential service industries. The majority of immigrants in Qatar originate from countries where low socio-economic levels and inadequate medical care are common, particularly from the Indian Subcontinent, Southeast Asia and Africa, and many are known to carry patent intestinal helminth and protozoan infections on arrival in Qatar [1]. In so far as transmission may be possible in Qatar, this presents a potential health hazard to the local community [2,3], especially if the arrivals find work in jobs associated with the preparation of food for public/domestic consumption. In this context, the extent of intestinal parasitic infections among two groups of immigrant workers employed in jobs associated with the food industry, food handlers and housemaids, from different geographical regions of origin was reported by Abu-Madi et al. [1]. However, little is known about the persistence of infections among long-term residents, and immigrants who have settled in the country.

In a broader context, surveys have been conducted in other countries in the region, focusing on the contrasting prevalence of intestinal parasitic infections among resident and immigrant sectors of populations. Approximately one-third of the population in Riyadh has been reported to be infected by intestinal parasites, and among these *Giardia duodenalis *and *Entamoeba histolytica *were the most common species, indicating the importance of parasitic infections in public health in Saudi Arabia [4]. In Kuwait, the highest rates of infection were found among Kuwaiti nationals followed by Bangladeshis, Sri Lankans, Indians and Egyptians, and these were attributed to importation of parasitic diseases by foreign labourers among whom *G. duodenalis *and *Trichuris trichiura *were the commonest, followed by *Strongyloides stercoralis *and *Hymenolepis nana *[5]. In Parma, Italy, 31% of foreign patients were affected by intestinal parasitosis [6] and more recently in Karaj city, Iran, analysis of the prevalence of intestinal parasitic infections among refugees and residents showed that parasitic infections were present (prevalence = 4.7%) and marginally higher among males compared with females [7].

Few studies have been conducted with respect to parasitic infections among immigrants to Qatar [1, Zainal, unpublished study] and we are not aware of any comparative studies contrasting prevalence among settled immigrant workers with residency permits to that among local long-term residents, the native Qataris. The recent work of Abu-Madi et al. [1] has shown that many immigrant workers arriving in Qatar carry the three major soil-transmitted nematodes (*Ascaris lumbricoides*, *T. trichiura *and hookworms) and a range of intestinal protozoa (*E. histolytica/dispar*, non-pathogenic amoebae, *Blastocystis hominis *and *G. duodenalis*). Approximately 33% of arrivals were found to be infected with one or a combination of these species, but prevalence changed over the 2-year period during which arriving female subjects were monitored. This significant between-year effect is important because it emphasized the possibility of trends that could be sustained over a longer time frame, and unless such changes in prevalence are monitored over subsequent years, their relevance to longer-term epidemiological patterns will remain uncertain. Abu-Madi et al. [1] also found that the region of origin was a major determinant of the sort of parasites and the intensity of infection that were carried into the country on arrival by new immigrants destined for jobs as housemaids and food handlers. In contrast to the data now available for new immigrants (who are routinely screened within one week of arrival, a condition of permission to stay and legally enforceable), comparable information about the prevalence of infections among long-term residents in Qatar is extremely limited. However, data on prevalence of parasitic infections among native residents is equally important because such data should form the baseline against which infections among immigrants can be assessed, and this is regarded as a major gap in our knowledge about parasitic infections in the populations living in Qatar. The key issues for this study are then whether there are consistent long-term upwards trends in prevalence of particular parasitic infections among settled immigrants and whether these are disproportionally evident among arrivals from particular regions/countries. In this paper we address these gaps in knowledge by assessing over a longer period than has been studied to date (4 years) both intestinal helminth and protozoan infections among native residents and immigrants who are resident. Our results raise some concerns with regard to public health in Qatar, and we consider their implications in the discussion.

## Methods

### Study subject and Sample collection

Doha is the capital city of the state of Qatar, located on the Arabian Gulf, having a population of about 1.8 million in 2007 and consisting of administrative, commercial, industrial and residential areas, with patches of agricultural land that are mostly grass fields.

The subjects for this study were 9208 individuals from different departments of Hamad Medical Corporation (HMC) hospitals including maternity, paediatrics, internal medicine and gastroenterology who had been referred for a routine stool test. The present study was based on a retrospective survey of intestinal parasitic infections from the records held at HMC data-base (MediCom) maintained at the Microbiology Laboratory at HMC and its outpatient clinics between 2005 and 2008. Ethical approval for access to these data was obtained from the Medical Research Centre and Research Committee at HMC, Qatar (Research protocol # 8060/09). Faecal samples were obtained from subjects referred for examination at HMC as part of a routine screening policy for the diagnosis of diseases associated with intestinal infections. Confidentiality was maintained throughout and the identity of subjects was not available to us, other than through each individual reference number. Age, sex and geographical region were recorded for each patient prior to taking the specimen. Fresh faecal specimens were collected in 25 ml clean wide-mouth, covered plastic containers. Stool samples were then immediately transported to the Microbiology Laboratory at HMC.

### Stool examination

Stool examination was carried out in a safety cabinet, where stool specimen was preserved in an ecofix preservative vial (Meridin Biosciences, Inc.). The contents were stirred with fine clean disposable wooden sticks to remove large clumps and mixed vigorously by vortex to homogenize the sample. To ensure adequate fixation of the homogenized stool, the sample was kept for half an hour at room temperature. The preserved specimen was mixed by vortex and filtered through a macro-con filtration unit for the removal of bulky debris. After filtration, 10% formalin and ethyl acetate were added; the sample was centrifuged for 10 min at 3000 rpm and the fluid containing diethyl ether and formalin was discarded. The pellet was re-suspended by agitation, poured onto a microscope slide containing one drop of iodine and examined microscopically for the presence/absence of parasite eggs/cysts and to enable identification of parasites in positive samples. Amoeba species other than *E. histolytica/dispar *including *E. coli*, *E. hartmanni*, *Endolimax nana *and *Iodamoeba buetschlii *were pooled together and recorded as non-pathogenic amoebae because the cysts were indistinguishable as described by Abu-Madi et al. [1]. We refer to *Giardia duodenalis *(= *lamblia/intestinalis*).

### Definition of variables

All birth dates and examination dates were recorded meticulously and the ages of subjects were classified into ranges by years. Thirteen age classes were then constructed to span ≤ 1 year, 1.1-1.9, 2.0-4.9, 5.0-9.9, 10.0-14.9, 15.0-19.9, 20.0-29.9, 30.0-39.9, 40.0-49.9, 50.0-59.9, 60.0-69.9, 70.0-79.9 and < 79.9 years.

The subjects in this study came from 28 countries. For the purpose of analysis, the subjects were grouped into four geographical groups for comparison with Qatari nationals (n = 3310). These were as follows (number of subjects is given in the parenthesis):

Arabian Peninsula (n = 511): Bahrain (40), Oman (65), Saudi Arabia (98), United Arab Emirates (21) and Yemen (287).

Eastern Mediterranean (n = 874): Iraq (77), Jordan (342), Lebanon (67), Syria (142) and Palestine (246).

Africa (n = 1299): Algeria (21), Egypt (719), Eritrea (11), Ethiopia (37), Gambia (2), Ghana (3), Somalia (54), Sudan (401) and Tunisia (51).

Asia (n = 3214): Afghanistan (3), Bangladesh (348), India (1049), Indonesia (39), Iran (173), Nepal (507), Pakistan (697), Philippines (230) and Sri Lanka (168).

### Statistical analysis

Prevalence data (percentage of subjects infected) are shown with 95% confidence limits, calculated as described by Rohlf & Sokal [8] employing bespoke software. Prevalence was analyzed by maximum likelihood techniques based on log linear analysis of contingency tables using the software package SPSS (Version 16.0.1.). Initially, full factorial models were fitted, incorporating as factors sex (2 levels, males and females), age (13 levels as shown in Table 1), year of study (4 levels, 2005, 2006, 2007 and 2008) and region of origin (5 levels, Africa, Arabian Peninsula, Asia, Eastern Mediterranean and Qatar). Infection was considered as a binary factor (present/absent). These explanatory factors were fitted initially to all models that were evaluated. For each level of analysis in turn, beginning with the most complex model, involving all possible main effects and interactions, those combinations that did not contribute significantly to explaining variation in the data were eliminated in a stepwise fashion beginning with the highest-level interaction (backward selection procedure). A minimum sufficient model was then obtained, for which the likelihood ratio of *χ*2 was not significant, indicating that the model was sufficient in explaining the data (these values are given in the legends to the figures as relevant). The importance of each term (i.e. interactions involving infection) in the final model was assessed by the probability that its exclusion would alter the model significantly and these values relating to interactions that included presence/absence of infection are given in the text. The remaining terms in the final model that did not include presence/absence of infection are not given but can be made available from the authors on request.

**Table 1:** No of subjects in each category and the prevalence (%) of the four species of helminths by age, sex, region and year

		No Subjects	Hookworms	*T. trichiura*	*A. lumbricoides*	*H. nana*	Combined
**Age class Age (years)**							
1	0-1	140	0	0	0	0	0
2	1.1 - 1.9	575	0	0	0.2	0	0.2
3	2.0 - 4.9	1084	0.1	0	0.1	0.1	0.3
4	5.0 - 9.9	903	0	0.1	0	0	0.1
5	10.0 - 14.9	423	0.2	0	0	0	0.2
6	15.0 - 19.9	297	1.0	0.3	0	***0.7***	1.7
7	20.0 - 29.9	1377	7.0*	***1.7***	***0.9***	0.3	***8.6***
8	30.0 - 39.9	1345	4.6	0.6	0.7	0.1	5.3
9	40.0 - 49.9	1259	1.4	0.6	0.6	0	2.3
10	50.0 - 59.9	835	0.5	0.4	0	0.1	1.0
11	60.0 - 69.9	521	0.6	0.4	0	0	0.8
12	70.0 - 79.9	336	0.3	0	0	0	0.3
13	> 79.9	113	0	0	0	0	0
**χ^2^**			**158.6**	**32.6**	**24.3**	-	**
***P***			**< 0.001**	**0.001**	**0.018**	**NS**	
Sex							
	Males	5327	***3.4***	***0.7***	***0.5***	0.1	***4.2***
	Females	3881	0.2	0.2	0.1	0.1	0.4
**χ^2^**			**54.0**	-	-	-	**
***P***			**< 0.001**	**NS**	**NS**	**NS**	
Region							
	Africa	1299	0.2	0.1	0.1	0.2	0.5
	Arabian Pen.	511	0	0	0	0.2	0.2
	Asia	3214	***5.7***	***1.4***	***0.9***	0.2	***7.2***
	Eastern Med.	874	0	0	0	0	0
	Qatar	3310	0.1	0	0	0	
**χ^2^**			**154.5**	**62.0**	**40.4**	**10.4**	**192.4**
***P***			**< 0.001**	**< 0.001**	**< 0.001**	**0.035**	**< 0.001**
Year							
	2005	2559	1.0	0.3	0.2	< 0.1^+^	1.3
	2006	2120	1.3	0.2	0.3	< 0.1^+^	1.7
	2007	2220	***3.1***	0.7	***0.5***	0	3.7
	2008	2309	3.0	***0.8***	0.3	***0.3***	***4***
**χ^2^**			**38.8**	**8.7**	-	**11.7**	**48.1**
***P***			**< 0.001**	**0.034**	**NS**	**0.008**	**< 0.001**

*The highest prevalence in each category is in italics for emphasis.

+ In these cases there was just one infected subject.

** In this case see text for significant interactions between sex and age.

The statistical outputs were derived from minimum sufficient models, after first fitting all factors into a single full factorial model, and the degrees of freedom can be calculated from the number of levels within each factor minus 1. *χ*^2 ^values are not shown for non -significant terms because these had been removed from the model during backward elimination in the derivation of the minimum sufficient model. The *χ*^2 ^values for goodness of fit of the minimum sufficient models for hookworms, *T. trichiura*, *A. lumbricoides *and *H. nana *was as follows: 399.1 (dof = 838, *P *= 1), 368.9 (dof = 839, *P *= 1), 369.1 (dof = 842, *P *= 1) and 368.3 (dof = 851, *P *= 1), respectively. Additional terms in the final models, that did not incorporate the presence/absence of parasites are not shown, but can be made available on request from the authors.

## Results

The study comprised tests on 9208 subjects across 4 years, and Table 1 shows the number of subjects by each of the primary factors analysed (main effects). Of these 943 (10.2%) were infected with one or more of the parasites in the study and overall prevalence of each of the species in the study is given in Table 2.

**Table 2:** Prevalence (%) of protozoan and helminth parasites in the study population. (The values are based on the 9208 subjects that were screened for this study)

	No. Infected	Prevalence (%)	95% confidence intervals
**Protozoa**			
*Blastocystis hominis*	398	4.3	3.91 - 4.74
*Giardia duodenalis*	179	1.9	1.66 - 2.23
Non path amoeba*	232	2.5	2.20 - 2.84
Path amoeba*	27	0.3	0.08 - 0.43
All protozoa combined	735	8.0	7.43 - 8.54
			
**Helminths**			
Hookworms	189	2.1	1.76 - 2.34
*Trichuris trichiura*	45	0.5	0.16 - 0.65
*Ascaris lumbricoides*	31	0.3	0.01 - 0.48
Hymenolepis nana**	9	0.1	0.05 - 0.19
All helminths combined	242	2.6	2.30 - 2.95
			
All the above species combined	943	10.2	9.62 - 10.86

* Non path amoebae are non pathogenic amoebae, and path are pathogenic amoebae. See text for further explanation

**This species is also known as *Rodentolepis nana*

### All parasites combined

**Figure 1:**
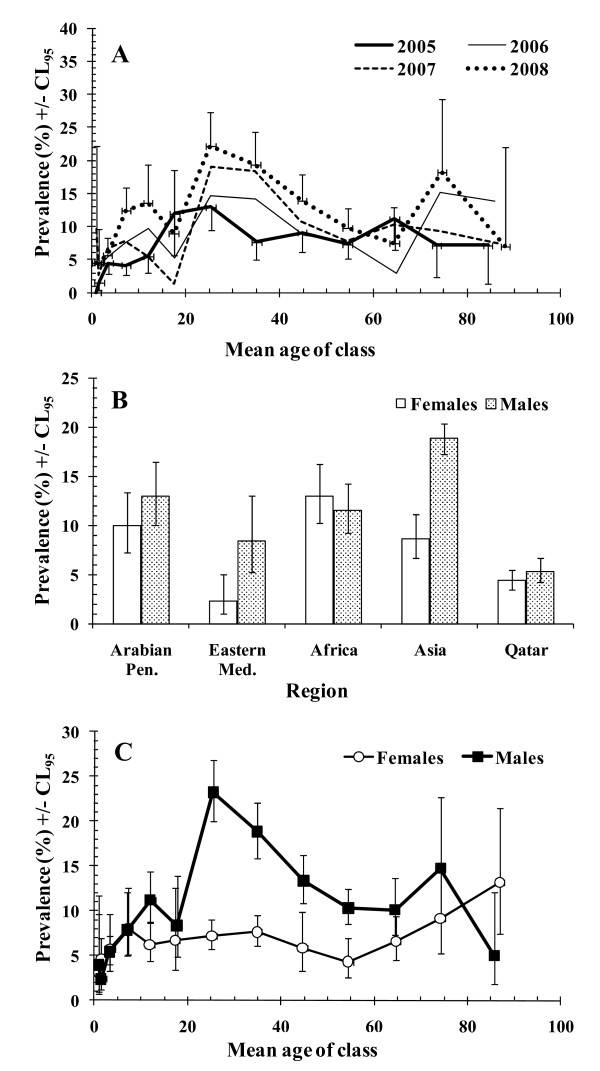
**Factors affecting the prevalence of all parasitic infections combined**. The significant terms in the minimum sufficient model are illustrated in (A-C) and the statistical significance is presented in the text. The model also included two expressions which did not encompass presence/absence of parasites and these are not given. For the goodness of fit of the minimum sufficient model, the likelihood ratio *χ*^2^_786 _= 746.5 (*P *= 0.84). A. Changes in prevalence by age class and year of study. The number of subjects in each age class varied from 22 - 402 and see Table 1 for further details. The 95% confidence limits are only shown on two data-sets to give an idea of the range and to avoid obscuring the data. B. Effect of sex on prevalence across the 4 regions from which immigrant workers originated and native Qataris. The number of subject in each group was as follows from left to right for females = 500, 271, 833, 396, 1881 and for males 799, 240, 2381, 478, 1429. C. Prevalence of infection in both sexes by age class. The number of subjects in each age class varied from 53 - 516 among females and from 60 - 861 among males.

When all the parasite species were combined, a significant steady increase in prevalence was detected across the 4 years of the study (2005 = 7.8% [6.74-8.81], 2006 = 9.3% [8.10-10.58], 2007 = 10.6 [9.35-11.91] and 2008 = 13.4% [12.04-14.82]), almost doubling over this period. However, this increase over the years was confounded by an interaction with age (year*age class*presence/absence of infection, *χ*^2^_36 _= 52.5, *P *= 0.037) and this is illustrated in Figure 1A. The increased prevalence with year of study here is particularly apparent in the middle aged subjects (in age classes 7-9, corresponding to subjects 20.0 to 49.9 years old) but also among some, but not all, of the other age classes as well.

Variation among regions of origin of immigrants and the local Qatari residents was dependent on sex (sex*region*presence/absence of infection, *χ*^2^_4 _= 25.0, *P *< 0.001) and this is illustrated in Figure 1B. The greatest discrepancy in terms of prevalence between sexes was among Asian workers, where prevalence was more than twice (× 2.18) as high in males compared with females. An even greater relative discrepancy in favour of males was found among workers from the Eastern Mediterranean region (× 3.68) although the prevalence in both sexes was lower compared to that among Asian workers. Discrepancies in prevalence between sexes were far less marked among those from Africa, the Arabian Peninsula and among Qataris. Among males (Figure 1B) prevalence was lowest in Qataris and highest among Asian immigrants, with little difference between immigrants from the other three regional groups. Among females prevalence was lowest in subjects from the Eastern Mediterranean, somewhat higher among Qataris, but substantially higher among women from Africa, Asia and the Arabian Peninsula.

Figure 1C shows how prevalence varied with age among the two sexes (sex*age class*presence/absence of infection, *χ*^2^_12 _= 27.8, *P *= 0.006). As can be seen, prevalence was generally low among females (< 10%) until age over 60, and then steadily increased among the oldest members of the community. In contrast among males, prevalence rose to over 20% in the 20.0-29.9 aged subjects and then fell steadily with age, falling lower than in females in the oldest subjects (> 80 years old).

### All helminths combined

Helminth infections were rarer than protozoan infections; only 242 subjects (2.6%) were recorded as carrying these parasites (Table 2). Hookworm infections were most common (prevalence = 2.1%), and the others were all lower (Table 2). Three subjects carried 3 species of helminths, 26 had double infections and the remaining 213 all had one of the 4 species that were detected. Analysis of prevalence of helminth infections revealed a very substantial regional effect (*χ*^2^_4 _= 192.37, *P *< 0.001, Table 1). Most of the 242 positives (95.5%, *n *= 231) were from the Asian region. Among this group 3214 subjects had been examined and the prevalence of helminth infections was 7.2%. Numerically and in terms of prevalence most cases came from Nepal (*n *= 115, prevalence among the Nepalese = 22.7%), then Sri Lanka (9.5%), Bangladesh (7.2%) and India (5.4%). There were 7 cases among subjects from Africa and 3 among Qataris. Regions with extremely low prevalence included the Arabian Peninsula and Eastern Mediterranean, with only 1 subject (from Oman) infected with *H. nana *of 511 subjects from the Arabian Peninsula and none of 874 subjects from the Eastern Mediterranean region with evidence of helminth infection.

Overall the prevalence of helminth infections jumped, more than doubling, between 2006 and 2007 (Table 1) and this increase was significant (year*presence/absence of infection, *χ*^2^_3 _= 48.1, *P *< 0.001). When the analysis was confined to subjects from Asia, prevalence by years (2005-2008) rose from 3.7% to 4.7%, then to 10.1% and 9.7%, respectively.

The vast majority of infections (93.0% of the individuals carrying worms) were male subjects (Table 1), but male sex was confounded by a weak but significant sex by age interaction (sex*age*presence/absence of infection, *χ*^2^_12 _= 21.1, *P *= 0.048). Examination of the age classes revealed that 90.1% of cases were confined to the 20.0-49.9 age range, with a peak among those aged 20.0-29.9 (Table 1, 48.8% of all helminth infections and 8.6% of this age class). There were only sporadic cases among younger individuals, the youngest infected child being just 1.4 years old (*A. lumbricoides*), but in total only four children carried worms in the under 5 age groups (n = 1799 children aged less than 5 in the study). At the opposite end of the age range, there were 4 cases of helminth infection in the 60.0-69.9 year age class and 1 in the 70.0-79.9 year age class.

So to summarise, helminth infections were mainly, although not exclusively, carried by male Asian subjects in the age range 20.0-49.9, and within this group 20.0-29.9 year-old male Nepalese immigrants contributed the most to helminth prevalence. Furthermore, the prevalence of helminth infections increased 3-fold between 2005 and 2008.

### Individual species of helminths

Not surprisingly, analysis of each of these species separately yielded much the same pattern as that above for the analysis of the combined data. Table 1 shows prevalence rates for each species in the different subsets of the data. Summarizing the outcomes of the analyses, all three nematodes were mainly encountered among subjects from Asia. All were more common among male subjects, with the exception of the cestode *H. nana*. With respect to age, peak prevalence of all three species of nematodes was in the 20.0-29.9 age class, and for *H. nana *it was in the 15.0-19.9 age class. The prevalence of the nematodes species jumped upwards between 2006 and 2007 but the cestode *H. nana *was primarily encountered in 2008. With the exception of the sex effect, and the age effect in *H. nana *all the other effects (region of origin and year of survey) were significant in all four species (statistics are shown in Table 1). The sex effect was only significant in the case of hookworms, with prevalence being 17 times higher in males compared with females.

### All protozoan infections combined

Protozoan infections were more common than helminth infections and the prevalence of individual species is given in Table 2. Altogether 735 subjects (8.0%) in the study were infected by at least one of the species tested.

**Figure 2:**
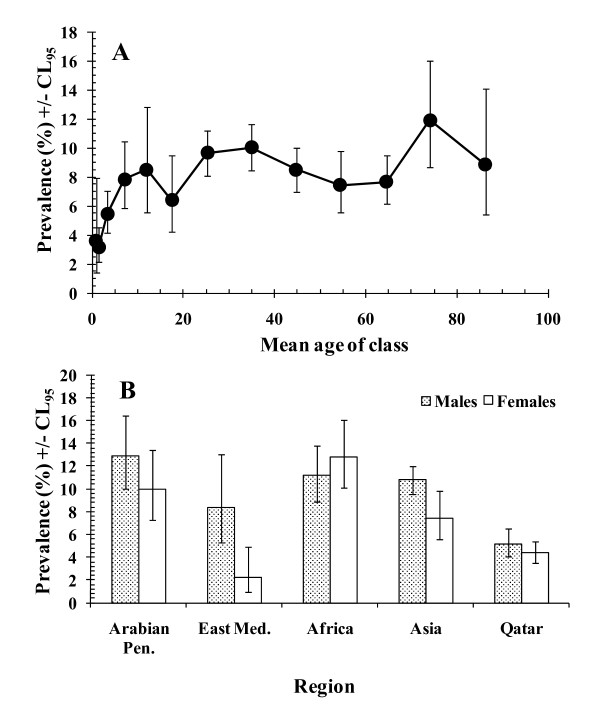
**Prevalence of all protozoan infections combined**. For the goodness of fit of the minimum sufficient model, the likelihood ratio *χ*^2^_834 _= 804.3 (*P *= 0.76). Pen. = Peninsula and Med. = Mediterranean. A. Prevalence by the age classes in the population. For numbers of subject in each age class see Table 1B. Variation in prevalence in male and female subjects across the 5 regions in the study. For numbers of subjects in each subset see legend to Figure 1B.

When species were combined, there was marked variation of prevalence of protozoan infections by host age (age*presence/absence of infection, *χ*^2^_12 _= 58.0, *P *< 0.001). Figure 2A shows that some infections were detected in all age classes and even among the youngest age class (children less than 1 year old) in which the prevalence was 3.6%. Prevalence then rose with age and stabilized by the time the children were about 5.0-9.9 years old, varying only in the range 6-12% right through to the oldest sectors of the sampled population.

As with the combined data for helminths, prevalence of protozoan infections increased with year of study. In 2005, prevalence was 6.6%, then 7.9%, 7.5% and in 2008 it was 10.0%, thus increasing 1.5-fold over the four-year period, and these differences between years were significant (year*presence/absence of infection, *χ*^2^_3 _= 14.9, *P *= 0.002).

Prevalence also varied, to different extents, between the sexes from the five regions from which subjects originated (region*sex*presence/absence of infection, *χ*^2^_4 _= 17.9, *P *= 0.001). Figure 2B shows that among subjects from the Eastern Mediterranean protozoan infections were much more common among male subjects (× 4.9). There was also a discrepancy between the sexes in favour of males among subjects from Asia, Arabian Peninsula and marginally so among Qataris, but among subjects of African origin the discrepancy was marginally in the opposite direction. When examined separately by sex in turn, prevalence among males was lowest in Qataris and highest in those from other states in the Arabian Peninsula, but only marginally lower among those from the Eastern Mediterranean, Africa and Asia. In women, prevalence was lowest among those from the Eastern Mediterranean, higher among Qataris and highest in those from Africa.

#### Individual species of protozoa

##### B. hominis

This was the commonest species of intestinal protozoan, with an overall prevalence of 4.3%, almost twice that of the next most common, the non-pathogenic amoebae (Table 2). The prevalence of *B. hominis *infections oscillated over the four-year period being lower in 2005 (3.6%, 2.86-4.37) and 2007 (3.6%, 2.90-4.53), than in the intervening years 2006 (4.5%, 3.67-5.53) and 2008 (5.6%, 4.69-6.57). This year effect was significant (year*presence/absence of infection, *χ*^2^_3 _= 13.6, *P *= 0.003).

**Figure 3:**
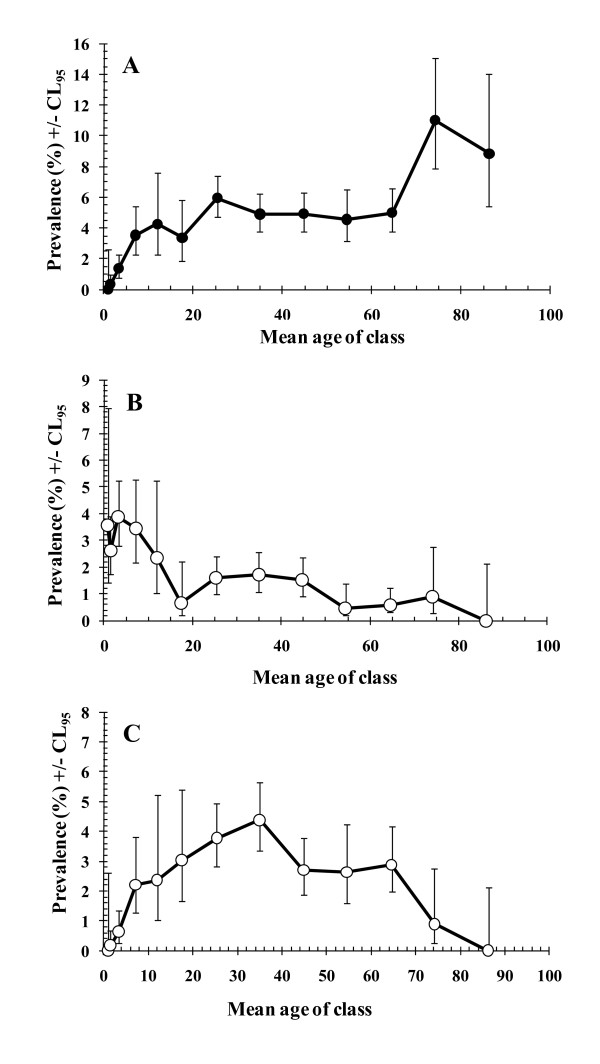
**Variation in prevalence of protozoan infections by age of host**. For numbers of subject in each age class see Table 1, and the statistical analysis of the individual components of the final model is given in the text. A. *B. hominis*. For the goodness of fit of the minimum sufficient model, the likelihood ratio *χ*^2^_834 _= 751.9 (*P *= 0.98). B. *G. duodenalis*. For the goodness of fit of the minimum sufficient model, the likelihood ratio *χ*^2^_842 _= 621.8 (*P *= 1). C. Non-pathogenic amoebae. For the goodness of fit of the minimum sufficient model, the likelihood ratio *χ*^2^_792 _= 577.3 (*P *= 1).

Prevalence increased among the youngest individuals to plateau in those in the 20.0-29.9 year age class (Figure 3A), and rose markedly again in those above 70 years old (age*presence/absence of infection, *χ*^2^_12 _= 128.6, *P *< 0.001), reaching over 10% in age class 12 (70.0-79.9).

There was no independent effect of region, with prevalence varying from 5.2% (4.46-6.00) among Asian immigrants to 6.1% (4.69-7.72) among those from the Arabian Peninsula. Although not independently significant, prevalence appeared lower among Qataris 2.8% (2.37-4.44). However, there was also a significant interaction between sex and region (sex*region*presence/absence of infection, *χ*^2^_4 _= 10.8, *P *= 0.029), but this was a relatively minor effect and was not investigated further.

##### G. duodenalis

**Figure 4:**
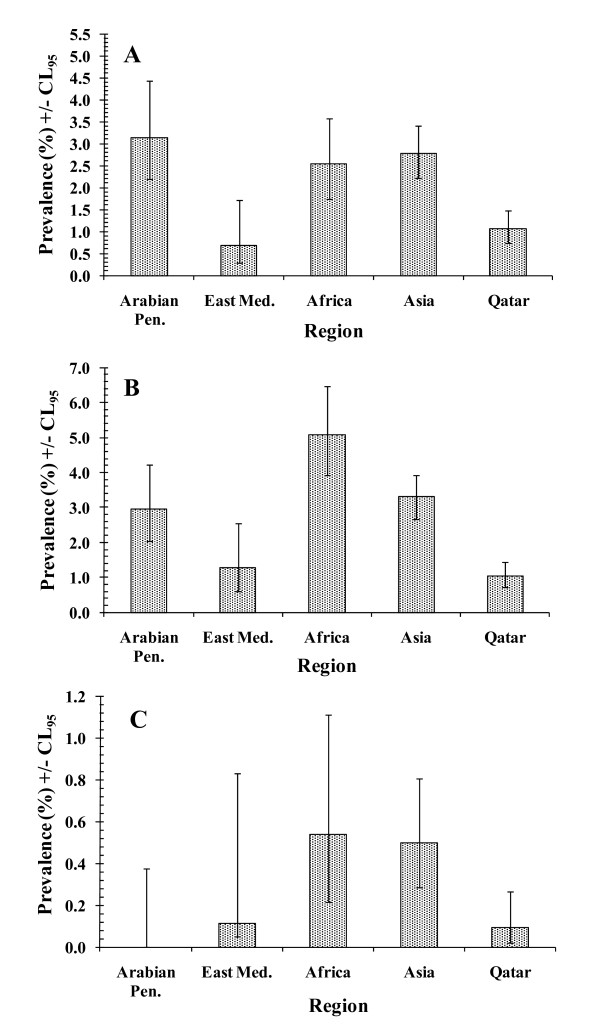
**Variation in prevalence of protozoan infections by region of origin of subjects**. For numbers of subject in each region see Table 1, and the statistical analysis of the individual components of the final model is given in the text. A. *G. duodenalis*. For the goodness of fit of the minimum sufficient model see legend to Fig. 3B. B. Non-pathogenic amoebae. For the goodness of fit of the minimum sufficient model see legend to Fig. 3C. C. Pathogenic amoebae. For the goodness of fit of the minimum sufficient model, the likelihood ratio *χ*^2^_842 _= 411.7 (*P *= 1).

Although the overall prevalence of *G. duodenalis *was 1.9%, there were significant differences between regions (region*presence/absence of infection *χ*^2^_4 _= 50.8, *P *< 0.001). As Figure 4A shows, prevalence of *G. duodenalis *was relatively low in Qatari subjects and those from the Eastern Mediterranean countries but was higher and very similar among subjects from the other three regions.

There was also a highly significant effect of age on the prevalence of *G. duodenalis *(age*presence/absence of infection *χ*^2^_12 _= 70.8, *P *< 0.001). In this case prevalence was higher among the younger age classes of the population (Figure 3B) and fell with increasing age.

##### Non-pathogenic amoebae

The prevalence of the non-pathogenic amoebae was subject to a complex interaction involving sex, year and region (sex*year*region*presence/absence of infection, *χ*^2^_12 _= 25.6, *P *= 0.012), and this was not explored further. Figure 4B shows the regional effect and this indicates that prevalence was highest among the subjects from Africa and lowest among the Qataris.

However, there was a strong effect of age (age*presence/absence of infection *χ*^2^_12 _= 65.6, *P *< 0.001), and as Figure 3C shows this was quite different to those for *G. duodenalis *and *B. hominis*. In this case a curve was evident with rising prevalence among the younger subjects, peak prevalence among the 30.0-39.9 year-old age class, and then a fall in prevalence among older subjects. The best fit polynomial curve was given by y = 0.4104 + 0.164x - 0.002x^2 ^(R^2 ^= 0.836), where y = prevalence and x is the age in years.

##### Pathogenic amoebae (E. histolytica/E. dispar)

There were only 27 subjects diagnosed as carrying pathogenic amoebae (Table 2). Analysis revealed only two simple effects. Prevalence differed significantly between regions (region*presence/absence of infection *χ*^2^_4 _= 13.4, *P *= 0.009). Figure 4C shows that no infections were found among the subjects from the Arabian Peninsula and very few among Qataris and those from the Eastern Mediterranean, and the highest prevalences were among those from Africa and Asia.

There was also a borderline significant effect of age (age*presence/absence of infection *χ*^2^_12 _= 21.7, *P *= 0.041). No infections were detected among children less than 5 years old and none among those over 70 years old. In-between these extremes prevalence varied from 0.1%-0.7%, with the maximum prevalence of 0.7% recorded among both the 10.0-14.9 and 50.0-59.9 year-old age classes. Since this species was largely concentrated among Asian and African immigrants (Figure 4C and 85.2% of cases), we re-analysed the age effect confining it to these two groups. This gave much the same outcome as the analysis among all regions except that among the combined Asian and African immigrants prevalence was 0.5% and among the age classes it ranged from 0% to 1.3%, with peaks among the 50.0-50.9 age group (1.3%), the 20.0-29.9 age group (0.8%) and the 10.0-14.9 age group (0.7%).

## Discussion

The data on which the analysis reported in this paper is based were derived from subjects examined in hospital for non-parasitic infections and/or non-related health problems, as well as routine health checks and included women in maternity wards. Therefore, they do not represent a totally random and completely unbiased selection of the population. Nevertheless, they did provide an opportunity to compare parasitic infections across the full range of age classes, right through from those less than a year old to individuals in their 80s, from both sexes, and from a range of nationalities. It is difficult to imagine how else such comprehensive data-set could have been obtained, given that invitation to provide stool samples from randomly chosen addresses in the city would have been subject to poor compliance for obvious reasons. The non-Qatari nationals were all domiciled in Qatar for shorter/longer periods of time, with a minimum residency of at least 5 years, with the obvious exceptions of those less than 5 years old who would have been largely second generation descendents of immigrants born in Qatar. We assume that the Qataris were long-term native residents, descendents of the tribes which originally settled the peninsula (since Qatari citizenship is mostly restricted to this group). So, within these limitations, we believe that the analysis revealed an interesting and realistic picture of how intestinal parasitic infections are distributed across the population as a whole, and how that picture has been changing in recent years. We were particularly interested in identifying high-risk groups within our study group who through harbouring most of the infections (high prevalence) may have been primarily responsible for the observed overall prevalence and represented a risk to the rest of the community in so for as they constituted reservoirs of infection. Once identified, they could be the targets for focused treatment. In respect of these objectives, our analysis revealed several important findings.

Firstly, the prevalence of helminths was generally reassuringly low (2.6%), and most infections were attributable to hookworms, the other soil-transmitted species being considerably rarer (< 0.5%). The possible explanation for these findings is twofold. Firstly the large expatriate population coming to work in Qatar from various parts of the world, but predominantly from the Asian subcontinent, undergoes pre-employment checkups and individuals are treated if found infected with helminths. Secondly, the climatic conditions of Qatar with hot temperatures (reaching to 50°C at times) are not suitable for the survival of the infective stages of these parasites. However, there was one clear risk group: young adult male subjects from Nepal, where the prevalence of all three GI helminths, and especially hookworm, is known to be moderate to high in rural populations (e.g. 13.0 - 83.3% [9-14]), accounted for 47.5% of all helminth infections (115/242 positives) and among them 22.7% were infected with worms. Amongst the Asian expatriate populations the Nepalese are generally considered to be the most economically backward and undernourished [15,16]. Moreover, they constitute the bulk of the unskilled cleaner class of workers in Qatar. This can be substantiated by the fact that tuberculosis and enteric bacterial diseases in Qatar also seem to predominate in workers from Nepal (Doiphode, unpublished observations). Most of the remaining cases came from Asian immigrants. In this respect, our study has helped to identify two risk groups for harbouring helminth infections in Qatar: Asian immigrants and especially young adult males from Nepal. If the prevalence of helminths in Qatar is to be reduced, subjects from these groups should be targeted with anthelminthic treatment as a priority. There is probably little risk to the general population of Qatar from these carriers of hookworm infections because these infections are more likely to have been acquired abroad than in Qatar, where the hot, mostly arid climate (humidity is high only in July to September) is not conducive to the survival in the environment of hookworm eggs and the free-living stages of this parasite that ultimately develop into infective larvae. Nevertheless, this aggregation of helminth infections among Asian immigrants warrants some explanation. Intuitively, immigrants from Africa might also have been expected to harbour helminths, but only 7 such cases were recorded among the 1299 subjects from Africa that were screened in our survey. It is possible that subjects from Asian countries, such as Nepal, return home to their families more regularly than other immigrant groups, and hence they are exposed to repeated infections during such visits. Other immigrant groups may be more settled in Qatar and less likely to return to their native communities, quite as frequently, or simply dwell in living conditions in Qatar that enable helminth infections to be transmitted locally despite the generally inhospitable climate that otherwise does not support transmission of human helminth infections. We have no quantitative supporting evidence for either of these possibilities.

Secondly, whilst previously (2005-2006) we had observed a fall in the prevalence of helminths among recently arrived female food handlers and maids, among residents in the present study, the data show little change between 2005 and 2006 and then by 2007 demonstrate an increased overall prevalence of helminths and in each of the constituent species, hookworms, *T. trichiura *and *A. lumbricoides*. The higher prevalence levels noted in 2007 were largely maintained in 2008 at the entire study sample level, but closer scrutiny again indicates that prevalence rose each year in the 20.0-29.9 age group (see for example Figure 1A), and these were mostly people of Asian origin. So the pertinent question again is why? One possible explanation is that deteriorating conditions in their home countries have resulted recently in enhanced transmission and exacerbation of human helminth infections. Nepal in particular has been experiencing political unrest and economic hardship in recent years. Alternatively, as indicated earlier, it may be that their living conditions in Qatar have deteriorated over this period, perhaps as a result of the workforce increasing with resultant overcrowding in their living quarters.

Overall, protozoan infections were more common than helminth infections, but here again prevalence increased by year, showing obvious upward trends across the study period, both for combined protozoan infections and for each species individually. These observations raise concern as to why the health services have failed to detect this trend through surveillance and equally why they have not implemented effective treatment. The data indicate that no national group was free from infection with intestinal protozoa, although women of East Mediterranean origin and both Qatari sexes had relatively low prevalence. In this case, in addition to being acquired during home visits abroad, transmission is more likely to have been reinforced locally among people living in Qatar through contaminated water and food resources [17].

When the intestinal protozoa were pooled the age-prevalence curve showed rising prevalence in childhood and then stability at about the 10% level across most of the other age classes (Figure 3A). However, this hid three underlying and quite contrasting age-prevalence curves for three of the contributing parasite taxa. Prevalence of *G. duodenalis *was highest among young children, that of *B. hominis *peaked among the older sectors of the community, while for non-pathogenic amoebae the peak was in the middle aged groups. So these three taxa showed clearly different age-related epidemiological patterns. All are transmitted by the faecal-oral route, through contaminated food and water, and hence these distinct patterns are more likely to reflect difference in host parasite interactions, rather than age-related differences in exposure, although the latter may also play some role [18,19].

The age-prevalence curve for *G. duodenalis *conforms closely to that typically reported for this species in populations where infections are endemic rather than epidemic. A rapid rise in prevalence in infancy, peaking among the 2-8 year age groups is typical and consistent with our findings (peak prevalence in age classes 3 [2.0-4.9 years] and 4 [5.0-9.9 years]). The peak among the young, followed by a declining prevalence, with a shallower gradient compared to that seen during the ascending phase, and persistence of low prevalence into the oldest sectors of the community appears fairly typical of this parasite in developing countries where transmission is constant [18,19]. *G. duodenalis *stimulates a protective immunity in a large proportion of individuals [20,21], resulting in most subjects experiencing self-limiting acute infections in childhood with an average duration of about 2 months, and this largely explains the peak prevalence among children followed by the decline in the teenage groups. However, in some individuals the parasites can persist for much longer [22], and the existence of these chronically infected carriers helps to sustain the low-level prevalence among the older sectors of the population. *G. duodenalis *infections are known to have their most severe consequences on health among children, especially in the first 3 years of life, so from the perspective of public health importance, it is pertinent that in our study age class 3 (2.0-4.9 years old) had the highest prevalence of this parasite [23].

In common with some other studies, *B. hominis *was the most frequently encountered protozoan in the present study [24-26], but in Qatar overall prevalence was low at 4.3%, compared to some other studies (20.2%-54.5% [26-28]). Initially, prevalence of *B. hominis *rose with age, but more slowly than for *G. duodenalis*, stabilized and then rose even more dramatically among the elderly. Our data, therefore, did not reveal a distinct peak as some other studies have shown: for example a peak incidence among the 30.0-39.9 year group presenting at an outpatient department of a hospital in Canada [29], or peak prevalence in 10-17 year-old subjects in communities where transmission was high (overall prevalence of 18.4-32.6%). Instead, a slower rise in prevalence is observed, peaking in the elderly (60 year-old group) when transmission is low (e.g. overall prevalence of 1.9% and 5.9% in two study sites in China, compared to 4.3% in our study) [27], just as in the current study. A peak among the elderly was also reported among patients seen at a hospital in Glasgow where the overall prevalence was also low [30] and in Papua New Guinea ([31] but see [24] for further references). Such peak prevalences among older age groups have been attributed to the peak shift [27], a phenomenon often reported among parasitic infections and characterised by peak prevalence shifting into older sectors of the community as overall prevalence of the pathogen, and hence the local transmission rate, declines [32]. Infections with *B. hominis *are often self-limiting in otherwise healthy individuals [33,34], although the range of duration of typical infections is not well known, possibly lasting for several years in untreated subjects [35,36]. Protective immunity to *B. hominis *is still a controversial topic but exacerbation in immunosuppressed subjects suggests that it does have a role to play in controlling the progression of infections [24]. If immunity to infection is slow to develop, then longer/repeated exposure will be necessary to induce immunity to challenge, and prevalence in a population will not decline as rapidly as when transmission rates are higher. However, this still leaves the question of why prevalence peaked so late, in those 60 years old and above [[27,30], our data]. Perhaps age-related infirmity, with resultant reduced vigilance in drinking and eating, and/or deteriorating immunocompetence, create this enhanced risk of infection among the elderly. If this is the explanation, then clearly it does not hold true for *G. duodenalis *and non-pathogenic amoebae which were rare among this sector of the population.

The pattern of age-related prevalence with non-pathogenic amoeba suggests increasing risk of infection among the very young, but slow development of immunity resulting in a peak in the 20.0-29.9 age groups and then a fall in prevalence among older subjects to an absence of infection among the very oldest subjects. In our study this taxon comprised several species which were not distinguished individually, and since prevalence of each is known to vary, with *E. coli *being generally the most common and *I. buetschlii *the rarest [19], the age-prevalence curve in Figure 3C comprises several overlapping curves each for one of the constituent species. Since untreated infection with *E. histolytica *can last for up to 2 years, it is likely that the others also show durations of a similar order. Moreover, if transmission levels are generally low, as our overall prevalence of 2.5% suggests, and infections with the individual species are acquired in series, and for the same reasons that were discussed above for *B. hominis*, it is not surprising that peak prevalence was in the middle-aged group of subjects, rather than among children. The older subjects would almost certainly have been exposed continuously to infective cysts throughout life, and by 70.0-79.9 years of age would have had opportunity to have experienced each of the species in turn or collectively in concurrent infections. Hence, the declining age-prevalence suggests eventual acquisition of virtually solid protective immunity in all individuals in the 70+ age groups. *E. histolytica/dispar *was rare in the current study, and peak occurrence among the middle-aged groups is consistent with a low rate of transmission, although infections are known to peak earlier where transmission is more intense [37].

Finally this study has drawn attention to the extent of intestinal parasitic infection in a population where foreign immigrant workers constitute a large proportion of the inhabitants of Qatar. Inevitably they bring parasitic infections with them on first arrival, but our study has shown that infections are also harboured by those domiciled in the city for 5 or more years. Molina et al. [38] reported that some intestinal parasites, especially hookworms and *G. duodenalis*, persisted in Asian refugees who had lived in the U.S.A. for up to two years after arrival and this despite the provision of chemotherapy on entry into the country. The helminth infections in our study were largely carried by Asian immigrants and were probably mostly acquired abroad either persisting from before their original arrival in Qatar or acquired subsequently on return visits to their homelands. In this respect they are not a major public health concern, because transmission of hookworm in particular is unlikely to be efficient in the climate of Qatar. However, the protozoan infections are of more concern. These data are not simply explicable by acquisition abroad, and are more compatible with the idea that transmission is taking place within the local population. Since the native Qataris show lower prevalence of all the species identified, it is more likely that transmission is occurring in the poorer quarters of Qatar where immigrant workers with residency permits aggregate. The health authorities in Qatar therefore should focus attention on these sectors of the population and seek ways to reduce further the transmission potential. The increase in prevalence over the four years of our study, whilst in real terms still representing a small percentage of the total population, should be addressed in order to avert further spread in the years ahead.

## Competing interests

The authors declare that they have no competing interests.

## Authors' contributions

MAAM conceived the study, collected the data, and wrote the manuscript. JMB analysed the data and wrote the manuscript. SHD contributed to the interpretation of the results.
